# Search for Inhibitors of Mycobacterium tuberculosis Transketolase in a Series of Sulfo-Substituted Compounds

**DOI:** 10.32607/actanaturae.15709

**Published:** 2023

**Authors:** I. V. Gushchina, D. K. Nilov, T. A. Shcherbakova, S. M. Baldin, V. K. Švedas

**Affiliations:** Lomonosov Moscow State University, Faculty of Bioengineering and Bioinformatics, Moscow, 119234 Russian Federation; Lomonosov Moscow State University, Belozersky Institute of Physicochemical Biology, Moscow, 119234 Russian Federation

**Keywords:** ischemia/reperfusion, brain, intravenous transplantation, mesenchymal stem cells, microvascular density, reactivity, perfusion

## Abstract

As a result of the computer screening of a library of sulfo-substituted compounds, molecules capable of binding to the active site of transketolase from *Mycobacterium tuberculosis *were identified. An experimental verification of the inhibitory activity of the most promising compound, STK045765, against a highly purified recombinant enzyme preparation was carried out. It was shown that the STK045765 molecule competes for the binding site of the pyrophosphate group of the thiamine diphosphate cofactor and, at a micromolar concentrations, is able to suppress the activity of mycobacterial transketolase. The discovered furansulfonate scaffold may serve as the basis for the creation of anti-tuberculosis drugs.

## INTRODUCTION

Tuberculosis treatment is based on long-term multicomponent chemotherapy and is often accompanied by the development of drug resistance in* Mycobacterium tuberculosis*. Because of this, the search for new molecular targets and the development of drugs that can selectively suppress the growth of mycobacteria are of utmost importance. An analysis of the genome of the H37Rv strain of* M. tuberculosis *made it possible to establish metabolic pathways the suppression of which can provide the basis for the development of new drugs. In particular, the pentose phosphate pathway and the associated enzyme transketolase (mbTK) are crucial [1, 2]. mbTK catalyzes the reversible transfer of a two-carbon fragment from a donor substrate (ketose) to an acceptor substrate (aldose). One of the mbTK substrates, ribose 5-phosphate, is used for the synthesis of the mycobacteria cell wall [3, 4]. In this work, we carried out a computer screening for the ability of sulfo-substituted compounds to bind in the mbTK active center and experimental verification of the inhibitory properties of the selected, most promising candidate.

## EXPERIMENTAL

The molecular model of mbTK for docking was obtained based on the 3rim crystal structure [4]. Hydrogen atoms were added taking into account the ionization of amino acid residues with the AmberTools 1.2 software, then their coordinates were optimized with the Amber 12 package [5], using the steepest descent and conjugate gradient algorithms. A library of sulfo-substituted compounds for screening was constructed based on the Vitas-M commercial set of low-molecular-weight compounds (https://vitasmlab.biz) using a substructure search for the sulfo group in ACD/SpectrusDB (https://www. acdlabs.com). The compounds were docked into the active site of the mbTK model using Lead Finder 1.1.16 [6]. The search region included the binding site of the thiamine diphosphate cofactor and the substrate [7]. Then, compounds capable of forming an electrostatic interaction with the Mg^2+^ ion, as well as other favorable contacts, were selected using a Perl script for structural filtration.

The recombinant mbTK protein was obtained using the pET-19b plasmid carrying the *Rv1449c *gene and the *Escherichia coli *strain BL21(DE3). Protein isolation and purification were performed as described previously [8, 9]. The activity of mbTK was measured by the coupled NAD^+^ reduction reaction, catalyzed by glyceraldehyde 3-phosphate dehydrogenase from rabbit muscles [10]. The reaction mixture contained glycylglycine (50 mM), dithiothreitol (3.2 mM), sodium arsenate (10 mM), magnesium chloride (2.5 mM), thiamine diphosphate (5 µM), xylulose 5-phosphate (140 µM), ribose 5-phosphate (560 µM), NAD^+^ (370 µM), glyceraldehyde 3-phosphate dehydrogenase (3 U), and the STK045765 inhibitor at various concentrations (0–1000 µM). The reaction was started by adding a solution of the mbTK apo form to the reaction mixture incubated in a thermostated cell at pH 7.6 and 25°C. The reaction rate was monitored as an increase in the optical density of the solution at 340 nm using a Shimadzu UV-1800 spectrophotometer.

## RESULTS AND DISCUSSION

The mbTK active site contains the cofactor thiamine diphosphate, as well as the Mg^2+^ ion [4]. The interaction of the pyrophosphate group with Mg^2+^ makes a significant contribution to the binding energy of thiamine diphosphate and is important for the design of mbTK inhibitors that were not reported prior to our study.

**Fig. 1:**
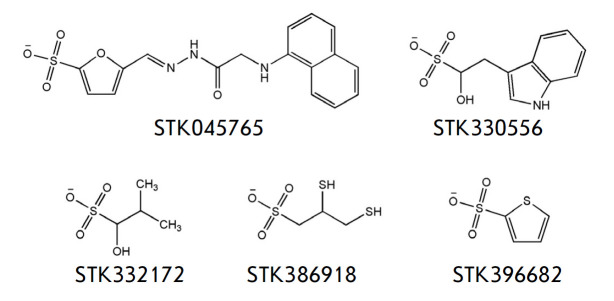
Chemical structures of potential mbTK inhibitors selected by computer screening

The sulfo group was chosen as a possible structural mimic of the pyrophosphate group capable of forming an electrostatic interaction with metal ions. From the library of commercially available low-molecularweight compounds, 320 molecules with a terminal (negatively charged) sulfo group and 563 molecules with an esterified sulfo group were retrieved. As a result of docking, the positions of compounds of this class in the mbTK active site were determined. The docking poses were further subjected to structural filtration by taking into account direct electrostatic interactions of the sulfo group with Mg^2+^. An expert analysis of the positions of the selected compounds identified five compounds with a terminal sulfo group (*Fig. 1*) that effectively interacted with Mg^2+^ and the surrounding residues of the mbTK active site. Less effective interactions of esterified sulfonates indicate that a negatively charged group is required for the inhibitor binding in the mbTK active site.

**Fig. 2:**
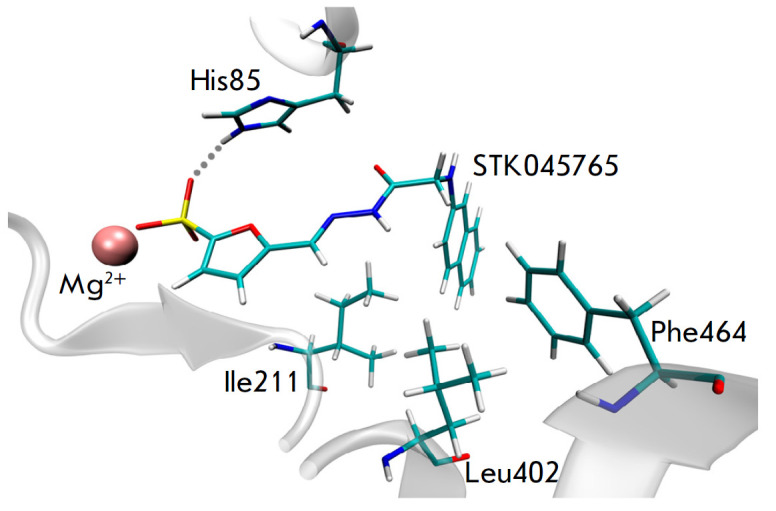
Model of the enzyme-inhibitor complex of mbTK and STK045765. The sulfo group is able to interact with the Mg^2+^ ion and form a hydrogen bond with the His85 residue; the hydrophobic bicyclic structural fragment is complementary to the site formed by the Ile211, Leu402, and Phe464 residues. The figure was prepared using VMD 1.9.2 [11]

**Fig. 3:**
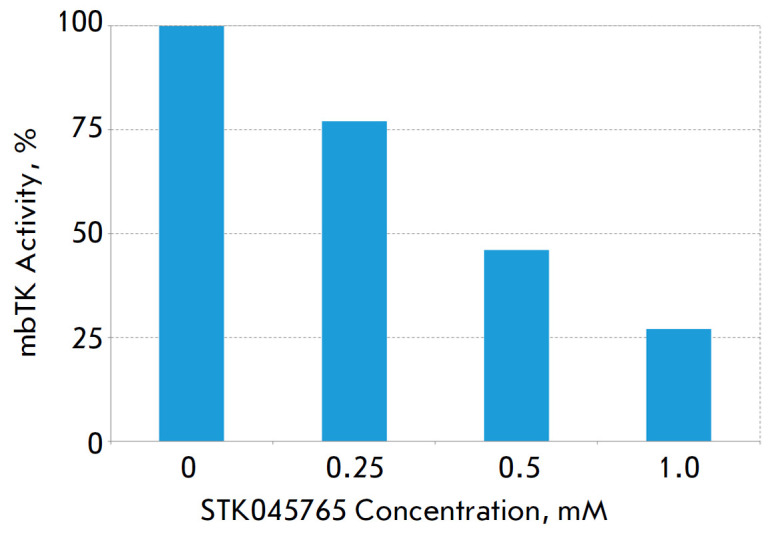
Effect of the STK045765 inhibitor on the catalytic activity of mbTK

For experimental testing of inhibitory properties, the STK045765 molecule was selected, which forms the most favorable bonds and contacts when modeling enzyme-inhibitor complexes. In this molecule, the furansulfonate and naphthalene fragments are connected by a hydrazide linker. The negatively charged sulfo group of STK045765 is able to interact with the Mg^2+^ ion and His85 side chain (*Fig. 2*) in a similar manner to the pyrophosphate group of the cofactor. Along with this, favorable hydrophobic contacts of the bicyclic structural fragment of STK045765 with the side chains of Ile211, Leu402, and Phe464 take place. Experimental verification confirmed the findings of molecular modeling. When an inhibitor was added to the reaction mixture, mbTK activity was suppressed: thus, in the presence of STK045765 at a concentration of 1 mM, the residual activity was 27% (*Fig. 3*).

## CONCLUSIONS

Virtual screening of a library of sulfo-substituted compounds made it possible to identify potential inhibitors capable of binding to the mbTK active site and competing with the cofactor thiamine diphosphate. Experimental testing of one of the candidates (STK045765 containing a furansulfonate group) against a highly purified mbTK preparation confirmed that compounds of this class are capable of inhibiting enzymatic activity. As a result of the study, the first-in-class inhibitor of mbTK was discovered, the structure of which can become the basis for the development of more effective inhibitors – prototypes of anti-tuberculosis drugs.

## Abbreviations

mbTK: mycobacterial transketolase
